# The cultural evolutionary trade-off of ritualistic synchrony

**DOI:** 10.1098/rstb.2019.0432

**Published:** 2020-06-29

**Authors:** Michele J. Gelfand, Nava Caluori, Joshua Conrad Jackson, Morgan K. Taylor

**Affiliations:** 1 Department of Psychology, University of Maryland, 1147 Biology-Psychology Building, College Park, MD 20742, USA; 2 Department of Psychology, University of Virginia, Charlottesville, VA 22903, USA; 3 Department of Psychology and Neuroscience, University of North Carolina, Chapel Hill, NC 27599, USA; 4 Department of Psychology and Neuroscience, Duke University, Durham, NC 27708, USA

**Keywords:** ritual, synchrony, trade-offs, culture, tightness–looseness

## Abstract

From Australia to the Arctic, human groups engage in synchronous behaviour during communal rituals. Because ritualistic synchrony is widespread, many argue that it is functional for human groups, encouraging large-scale cooperation and group cohesion. Here, we offer a more nuanced perspective on synchrony's function. We review research on synchrony's prosocial effects, but also discuss synchrony's antisocial effects such as encouraging group conflict, decreasing group creativity and increasing harmful obedience. We further argue that a tightness–looseness (TL) framework helps to explain this trade-off and generates new predictions for how ritualistic synchrony should evolve over time, where it should be most prevalent, and how it should affect group well-being. We close by arguing that synthesizing the literature on TL with the literature on synchrony has promise for understanding synchrony's role in a broader cultural evolutionary framework.

This article is part of the theme issue ‘Ritual renaissance: new insights into the most human of behaviours'.

## 1. Introduction

Over 100 years ago, in the Yaghan peninsula at the southern-most tip of South America, the Yamana people practised an elaborate initiation ritual. Young men brought the spoils of their hunt into the community's great hut where they shared it with other members of the village. Once the men had passed inside, those gathered around the hut began singing in harmony, and did not stop until the young men had shared their food with each of the hut's occupants and left [1]. Across the world in northern Australia, the Tiwi people engaged in a very different initiation ritual involving young men who jumped over a fire-pit. Before the fire jumping began, the jumpers circled the fire and danced using the same sequence of downward hand movements. Afterwards, the group danced in single file, chanting a song together [2].

These initiation rites are starkly different in many ways, but they do share a feature that recurs in societies around the world: ritualistic synchrony. Even though they were separated by tens of thousands of kilometres and their ancestry diverged thousands of years ago, the Tiwi and Yamana people each practised the same forms of synchronous dancing and singing, and they are far from alone. Ritualistic synchrony—including synchronous dancing, singing, chanting, drumming or marching—has been documented in every region of the world [3], and today it appears everywhere from choirs, to military parades, to pre-game rituals in rugby games [4].

Ritualistic synchrony's universality suggests that it may hold some kind of adaptive benefit for societies, as is the case with other global practises such as irrigation, tool use, cooking and children's games [5–7]. In particular, some have claimed that ritualistic synchrony increases social cohesion and cooperation in communities of humans [8–11], and even potentially in non-human animals [12].

The goal of our paper is to offer some nuance to this functionalist perspective. We review past research on the prosocial effects of ritualistic synchrony, and also summarize emerging research on the darker side of synchrony, including higher groupthink and destructive obedience and lower creativity. We then situate synchrony within broader literature on culture, suggesting that tightness–looseness (TL) theory provides a framework to explain this trade-off and to predict how ritualistic synchrony proliferates and evolves over time. Above all, by integrating synchrony with research on TL, we begin to understand synchrony's role in a broader cultural evolutionary framework.

## 2. The religious and intellectual history of ritualistic synchrony

Ritualistic synchrony has been practised by humans for thousands of years. Australian rock art dating back to 20 000–38 000 BCE appears to depict human figures beating sticks to the ground and engaging in synchronous dancing [13], and many of the world's oldest texts such as the Vedas, the Pyramid texts and the Book of the Dead depict synchronous marching, dancing or singing during ritual [14,15].

The intellectual study of synchrony, however, is far more recent. Religious ritual did not feature in many early theories of religion, which instead focused on the origins of religious beliefs rather than practises [16–19]. In the early twentieth century, Durkheim & Swain [20] reoriented the study of religion to focus on the *function* of religious practises, including ritualistic synchrony. Durkheim described this function by introducing the notion of ‘collective effervescence,’ the feeling of excitement and connection felt when a community participates in a collective or synchronous action. Religious rituals, Durkheim argued, fostered a sense of collective effervescence that was not only pleasant to experience, but was also adaptive for society as a whole. Durkheim theorized that participating in physiologically arousing and synchronizing religious rituals could be a bonding activity, leading people to feel more camaraderie with fellow participants and ultimately express more prosociality towards these participants. To the extent that religious rituals involved many members of a community, they could build valuable cross-cutting bonds within a society that increased cooperation and coordination.

This functionalist perspective has echoed throughout many more recent theories of religion [21,22]. Indeed, accounts of religion and morality [23,24], belief in supernatural punishment [25,26] and participation in doctrinal rituals [27] draw directly from Durkheim's argument that religion is adaptive for societies. As we discuss next, studies of ritualistic synchrony have taken a similarly optimistic view of synchrony's effects on human behaviour.

## 3. Empirical literature on synchrony: the good and the bad

### (a) The good: effects of synchrony on cooperation and cohesion

The study of synchronous rituals has been mostly experimental, allowing researchers to methodically test when and why ritualistic synchrony may promote cooperation and cohesion. While synchronous rituals observed in the field cannot be easily reproduced in the laboratory, experimental manipulations of synchrony often involve participants moving, dancing or vocalizing in synchrony with other participants in order to mimic the coordinated collective action of rituals. For example, participants in an experiment's synchrony condition may follow an experimenter while marching in step [8,9], tap to the same tune on a metronome [10,28,29], dance together while moving their limbs in the same way [30–34] or sing or chant together to the same tune [8]. By contrast, participants in the control group will engage in asynchronous actions, or actions with no explicit synchrony instruction.

Many of these experimental studies find that synchrony increases prosociality and cooperation compared to control conditions. Some studies have used economic games to show that synchrony increases people's tendency to make decisions that would maximize economic reward for the group, even at a potential cost to the individual [8–10,30,35,36]. For example, one study showed that chanting in sync increased people's donations to a collective pot of money, even though it was in their interest to withhold donations [10]. Other research has shown that synchronized participants are more likely to put effort into collective tasks instead of free riding [9]. Studies have even found that synchrony can increase costly altruism. Subjects who went through a synchrony manipulation were more likely to help when a co-participant in the synchronous activity became a victim of a moral transgression, even when helping was costly [37]. A number of these effects have been replicated within dyads [8,28,38] and large groups of over 40 people [9,39]. They have even been reproduced in analyses of real rituals. A study that examined nine rituals from different community groups in New Zealand found that those involving synchrony were the most likely to elicit group-beneficial decisions in economic games [35].

There has also been research on how synchrony affects self-reported group cohesion. Studies have found that people in a group that experiences synchrony tend to feel more trusting towards and united with their group members [8,10], feel more similar, and report more liking towards group members [28–30,37,40]. Synchrony also increases perceptions of social bonding [33,34,41–43], prosociality towards the ingroup [33,35] and the ability to get along with group members, even in difficult environments [32,39].

The prosocial effects of synchrony can be seen from a young age. Infants as young as 14–15 months expect social affiliation between synchronized actors [44] and are more likely to help an experimenter reclaim a dropped object after moving synchronously with the experimenter [11,45,46]. Synchrony also promotes prosocial behaviour towards peers in older children. For example, children who participate in synchronous activities together perceive themselves to be more similar [47] and are more helpful and cooperative towards each other [48,49] than children who do not experience synchrony. Children who moved in sync with each other are also more successful at completing joint tasks [50].

Some research suggests that synchrony's effects on group cohesion and trust could be potential mediators of the link between synchrony and cooperation [10,30,37]. Others have examined alternative mechanisms, such as enhanced attention and memory [51–53], mentalizing [54], viewing oneself and others as interdependent [55,56] and/or physiological changes that encourage feelings of group bondedness [30,38,57]. There is no consensus on which mechanism is most predictive of cooperation, and it is likely that these different mediators can coexist and simultaneously influence cooperation.

Synchrony not only affects cohesion, it also affects group potency. Military drills frequently involve synchronous marching and drumming in order to increase in-group bonds and make groups seem more formidable. These strategies appear to be effective: engaging in synchrony leads groups to overestimate their own formidability and to see their foes as less threatening [58]. Synchrony also makes groups appear more entitative [59,60], cohesive [61,62] and physically formidable [62] to outsiders. For example, when people see individuals waving in sync [60] or walking or speaking in sync [61], they perceive these individuals as more bonded.

These studies paint a clear picture of synchrony's prosocial effects. Not all studies have replicated these effects [41], and there are some conditions where synchrony does not help performance, such as in complex tasks where group members need to fulfil diverse and specialized roles [63,64]. Nevertheless, meta-analyses suggest that synchrony can increase cohesion, cooperation and coordination between group members [65–67]. Some studies even suggest that synchrony can have broad effects on prosociality that stretch beyond one's own ingroup to strangers and outgroup members [31,34,56,68]. Taken together, this research generally supports the view that synchrony is functional for communities.

### (b) The neglected dark side of synchrony: effects of synchrony on conformity and groupthink

The functionalist perspective on synchrony focuses exclusively on the benefits of synchronous rituals for groups. Improved cooperation and cohesion can indeed be adaptive for a group, lending the group an advantage in situations that would require coordination among its members. However, the major focus in the literature on synchrony's prosocial effects does not preclude the potential for synchrony to have a dark side.

In support of this notion, several studies have found that synchrony can promote conformity [40,69], aggression [70] and destructive obedience [71,72]. For example, studies on synchrony and conformity found that synchrony made people more likely to copy majority opinions when selecting products, rather than following their personal preferences [69]. Complementary studies on synchrony and destructive obedience found that synchrony—compared to a non-synchrony control activity—made people more likely to comply with a request to administer a sound blast to a stranger [71], and more likely to follow an experimenter's command to grind up live pill-bugs [72]. In these studies, synchrony promoted obedience, but to aggressive and morally compromised commands.

### (c) Does synchrony reduce creativity and productive dissent?

Building on this nascent work, we advance that synchrony presents a *trade-off* for groups that has been neglected thus far in the literature. While synchrony increases cohesion and cooperation, it may increase conformity, reduce creativity and foster groupthink. To explore this possibility, we conducted two studies which examined whether synchrony decreased groups' abilities to think creatively (study 1) and discouraged minority perspectives during a decision-making task (study 2).

Study 1 explored synchrony's adverse effect on group creativity, a relationship that had been raised in past literature [73,74], but never conclusively demonstrated. In this study, 149 participants assigned to 42 groups of either three or four individuals walked for 7–8 min around campus either in step with the experimenter (synchrony manipulation) or at their own pace (control condition). We chose this manipulation because it has been used in previous research to show that synchrony can facilitate cooperation [8,9] and formidability [58], and we wanted to test whether the same manipulation that spurs prosocial behaviour could also have detrimental effects on creativity. After the manipulation, participants wrote a collaborative story as a group, which two coders assessed for creativity and complexity. Coders were blind to condition when rating these stories (see the electronic supplementary material). We used latent profile analysis [75] to examine the effect of condition on story ratings while accounting for the fact that multiple participants contributed to the stories within each group.

**Figure 1.**
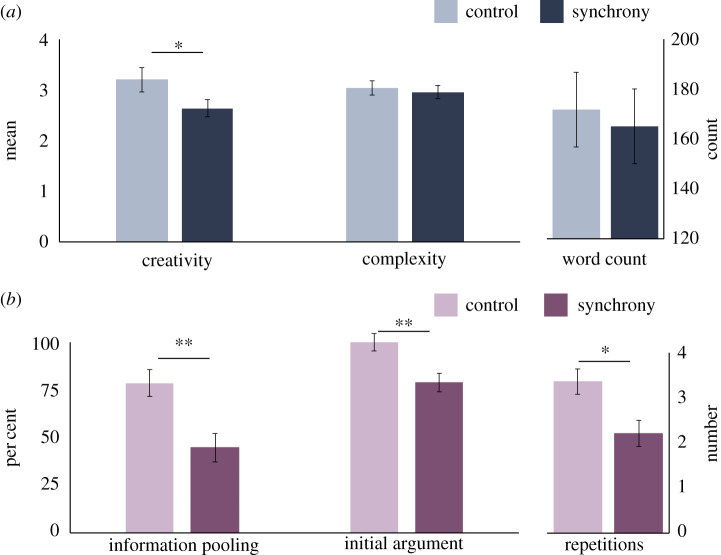
Synchrony's effects on creativity (*a*) and dissent (*b*). Error bars represent standard error. **p < 0.01, *p < 0.05.

We found that synchrony had the expected negative effect on creativity. Groups that had marched synchronously around campus wrote less creative stories than groups that marched at their own pace (table 1 and figure 1). Neither the complexity of stories nor the length of stories (word count) varied based on condition, demonstrating the unique effect of synchrony on suppressing creativity. Stories by synchronous groups showed more typical characters and less innovative storylines than stories by asynchronous groups, suggesting that coordination can in fact present roadblocks to group success when it requires creative thought.^1^

**Table 1.** Study 1 model statistics.

outcome	*b* (s.e.)	d.f.	*t*	*p*
creativity	−0.62 (0.29)	39	−2.11	0.04
complexity	−0.07 (0.19)	39	−0.34	0.73
word count	−9.04 (10.38)	39	−0.87	0.39

We next explored whether synchrony affects group dissent, testing whether synchrony would make participants less likely to speak out against their group even when it was in their group's interest. In this second study, 278 participants were assigned to 80 groups, each with three or four members. We manipulated synchrony through a chanting task adapted from past research [10] that required the group to either chant the same one-syllable words as each other (synchronous condition) or different one-syllable words from each other (asynchronous condition) for 6 min. We then measured group dissent using the ACME group decision-making task ([76]; see the electronic supplementary material). This task allowed us to measure the extent to which one member of each group who was randomly assigned to be given more complete information than other group members (termed the ‘minority participant’) spoke up to share information from their packet (termed ‘information pooling’), argued in favour of their opinion about which company to choose, and repeated these arguments. We predicted that, if synchrony suppresses healthy group dissent, these minority participants would be less likely to share their information and argue in favour of their unique opinions in the synchrony condition than in the control condition.

A *χ*^2^-test revealed that information pooling was significantly less likely in the synchrony condition than the control condition (table 2 and figure 1). A separate *χ*^2^-test also showed that fewer minority participants made an initial argument for their unique opinion in the synchrony condition than in the control condition. Minority participants in the synchrony condition also repeated their arguments fewer times than in the control condition after making an initial argument in favour of their unique opinion. Not only did synchrony suppress the initial urge to argue for one's unique opinion, it also suppressed the desire to *continue* to argue for one's own opinion, thereby reducing healthy group dissent.

**Table 2.** Study 2 model statistics.

information pooling	synchrony %	control %	groups	*χ*^2^	*p*
	44.7	78.6	80	9.74	0.002

initial argument	synchrony %	control %	groups	*χ*^2^	*p*
	78.9	100	80	9.82	0.002

argument repetition	synchrony *M* (s.d.)	control *M* (s.d.)	groups	*b* (s.e.)	*p*
	2.21 (1.80)	3.36 (1.82)	80	−1.15 (0.41)	0.006

These studies illustrate a darker side of synchrony that stifles creativity and individual thought within highly bonded groups. These effects could be hypothetically beneficial when groups need to make quick consensus-based decisions, but destructive when diversity and healthy disagreement are important for groups to make effective decisions [77].

## 4. Situating ritualistic synchrony within broader cultural evolutionary processes

### (a) What explains synchrony's cultural evolutionary trade-off?

**Figure 2.**
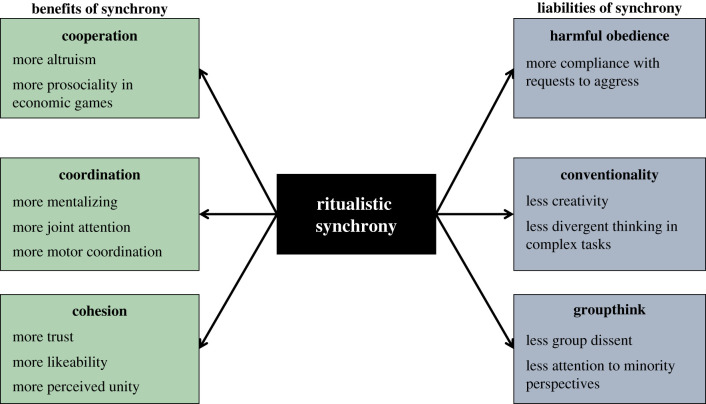
The proposed trade-off of ritualistic synchrony. (Online version in colour.)

The existing literature on ritualistic synchrony suggests a trade-off for human groups. On the one hand, synchrony leads people to feel closer with and more bonded to their group, and encourages group cooperation. On the other hand, synchrony also seems to increase people's obedience to aggressive and counterproductive group norms, and may decrease group creativity. Figure 2 summarizes this proposed trade-off.

There has been surprisingly little synthesis of synchrony's positive and negative effects. Elegant meta-analyses and reviews have discussed the prosocial [65–67] effects of synchrony, but few have integrated these effects with more negative effects of synchrony. Open questions thus remain about why synchrony has both negative and positive effects, which social ecologies might benefit most versus least from synchronous ritual, and whether synchrony shares features with other secular aspects of culture.

Here, we address these questions from the perspective of TL theory, a broad theory of how ecology gives rise to cultural and psychological variation. TL theory situates synchrony within a broader suite of features that emerge in societies to foster cooperation, cohesion and coordination at the expense of individuality and creativity, and identifies new directions for future research.

### (b) Tightness–looseness theory: a broad theory of cultural evolution

Around the same time that the literature on ritualistic synchrony was developing, another parallel literature on the strength of social norms, or TL, was evolving. TL theory suggests that all groups have social norms, but some groups' norms are tighter—with strict rules and punishments for deviance—whereas others are looser—with weaker rules and more tolerance for deviance. In this section, we illustrate how TL theory can address open questions about ritualistic synchrony.

TL theory has roots in ancient history and philosophy. Herodotus, an ancient Greek who is generally considered the father of history, contrasted the Persian openness to foreign ideas and practises with Egyptian rigidity around cleanliness, religion and authority relations [78]. Centuries later, Pelto [79] documented differences in the strength of norms across traditional societies, observing that the Hutterites, Hanno and Lubara were ‘tight’ in that they had strong norms, were very formal, and had severe punishments for norm violations. By contrast, the Kung Bushmen, Cubeo and Skolt Lapps were ‘loose,’ with weaker norms and more tolerance for deviance. Pelto speculated that these differences might arise from ecological conditions which forced communities to coordinate and cooperate, an intuition that was tested almost 50 years later by Gelfand *et al.* [80].

Gelfand *et al.* [80] found that differences in cultural tightness across 33 current-day nations could be traced to historical levels of natural disasters, disease prevalence, resource scarcity, and invasions. Later research demonstrated that variation across the 50 United States followed a similar pattern: compared to looser states, tight states had higher death rates owing to natural disasters, greater food insecurity, and more disease prevalence [81]. Jackson *et al.* [82] showed that non-industrial societies can also be differentiated on TL, and that ecological threats predict greater tightness. They also found that tightness is correlated with social complexity across cultures, perhaps because social complexity engenders a heightened need for the large-scale cooperation and coordination tightness can provide. While these studies were correlational, evolutionary game theoretic models have also shown that threat affects the evolution of tightness [83], and experimental research has shown that reminders of different threats temporarily tighten groups [84,85]. Neuroscience research using hyperscanning has likewise shown that coordination is higher under conditions of threat, at least in part owing to enhanced brain synchrony [86]. This line of research suggests that groups develop strong norms and punishments in order to coordinate to survive, whether owing to ecological and social threats or to increasing complexity and subsistence demands.

Research from TL theory is so relevant to the synchrony literature because tightness shares many of synchrony's trade-offs. Research on TL has also shown that as groups tighten to deal with coordination needs, they also experience a number of trade-offs associated with *order* versus *openness.* Tight groups have more monitoring, order, and self-control, which is critical for coordinating in the face of threat [81,87,88]. By contrast, loose groups that have fewer coordination needs are more open; they are much less ethnocentric and more tolerant of people from stigmatized groups [85], are more creative [81,89–91], and are more open to new ideas [92]. These symmetries between tightness and synchrony suggest that future research on the antecedents and consequences of synchrony may be able to fruitfully draw from existing research on TL.

### (c) Implications for regional and historical variation and trade-offs associated with synchronous ritual

Research on cultural tightness raises several new predictions for how synchrony may be distributed across cultures and how it may change over time. For example, one intriguing possibility is that ritualistic synchrony may also be most prevalent following periods of ecological and social threat, societal complexity, and subsistence styles that require coordination. While there has been little research on the role of threat and need for coordination in the evolution of ritualistic synchrony, there are a few suggestive studies. For example, in Malinowski's [93] ethnographic work in the Trobriand Islands, ritualistic synchrony was most common among groups who fished at sea, which was considerably more threatening than fishing in lagoons. Another study [3] found that larger, more complex groups had the highest levels of synchrony in their rituals. These studies suggest that ritualistic synchrony may have many of the same ecological correlates as cultural tightness.

TL theory helps us to understand why ritual may have positive and negative trade-offs in producing cooperation and cohesion at the expense of creativity and dissent, as the former two may be adaptive for dealing with threat and coordination needs while the latter two may not. TL research also raises intriguing possibilities for new research on the trade-offs of ritualistic synchrony. For example, does ritualistic synchrony relate to increased order, such as greater self-monitoring and higher self-control? Indeed, one study found that ritual improved children's ability to delay gratification [94]. On the other hand, like tightness, ritualistic synchrony may lead to lower openness, such as having more cultural inertia or resistance to change in groups. While our studies show how synchrony can decrease creativity and dissent in groups, future research could test whether synchrony—like tightness—increases ethnocentrism and the desire for autocratic leaders [85,90].

Other research on the TL trade-off is instructive for the ritualistic synchrony literature. One important question is how the intensity and frequency of rituals impacts group well-being. Recent work suggests that extreme levels of either tightness or looseness may be maladaptive. Harrington *et al.* [95] found a curvilinear effect such that nations with extreme tightness or looseness showed the lowest happiness relative to nations that are moderate on TL. Groups that are extremely loose may experience chaos and a lack of control and be unable to coordinate. By contrast, groups that are extremely tight may experience repression and a loss of any autonomy. This raises the question of whether there is an optimal level of ritualistic synchrony for groups.

Finally, future research could use the TL trade-off to explain the evolution of other religious beliefs and practises. For example, the belief in moralizing and punitive high gods shares many of synchrony's group-level effects. Moralizing religious belief predicts greater cooperation [96] and less cheating [97,98]. However, it also has a dark side, predicting aggression [99] and compliance to authority [23]. Recent studies even show that moralizing beliefs emerge during times of ecological threat and conflict [100,101], much like cultural tightness. This raises the intriguing possibility that ritualistic synchrony and moralizing high god belief serve many of the same cultural evolutionary functions, and may emerge in the same kinds of societies.

## 5. Conclusion

Many millennia have passed since the first human ritual, and many decades have passed since scholars began examining the potential function of ritualistic synchrony. Past research has examined ritualistic synchrony with rose-coloured lenses, documenting the positive effects of synchrony on cooperation and coordination. Here, we suggest that ritualistic synchrony represents a trade-off with both positive and negative effects on group behaviour. Synchrony may not only increase parochial cooperation and coordination, but may also increase obedience, groupthink and impair group creativity. By integrating this research with cultural tightness theory, we also raise the possibility that these trade-offs are adaptive to particular ecological and historical contexts where there is a need for coordination. This analysis situates research on ritualistic synchrony within a vast literature on cultural evolution.

## Supplementary Material

Supplemental Information for The Cultural Evolutionary Tradeoff of Ritualistic Synchrony

## Endnotes

1 We note that previous studies using this walking manipulation have used video recordings [9] and confederates [59] to confirm that the instruction to walk in synchrony does indeed produce synchronous movement. While we did not use one of these methods to confirm that movement, we believe that the success of these instructions in past studies lends credence to our manipulation's efficacy.

## Data accessibility

All data, analysis scripts, materials and coding manuals will be available at Open Science Famework (https://osf.io/amhr2/).
