# Supplementary material for: The cultural evolutionary trade-off of ritualistic synchrony
Source: Philos Trans R Soc Lond B Biol Sci. 2020 Jun 29;375(1805):20190432. doi: 10.1098/rstb.2019.0432 (PMC7423264; doi:10.1098/rstb.2019.0432)
Supplement: Supplemental Information for The Cultural Evolutionary Tradeoff of Ritualistic Synchrony [file rstb20190432supp1.docx]

**Supplemental Information for *The Cultural Evolutionary Tradeoff of Ritualistic Synchrony***

**Authors:** Michele J. Gelfand, Nava Caluori, Joshua Conrad Jackson, Morgan K. Taylor

**Journal:** Philosophical Transactions B

**Study 1**

**Participants & Methods**

In our first study, 149 undergraduate participants (55.9% female, *M*_age_ = 20.26, *SD*_age_ = 2.59) were assembled into groups of 3 or 4 (42 groups total). These groups were randomly assigned to either a synchrony or control condition. In both the synchrony and control groups the experimenter informed participants that they would be walking as a group around campus for 7-8 minutes. In the synchrony condition, participants were instructed to walk in step with each other the whole time, while in the control condition participants went on the same walk, but were given no walking instructions [1-2].

When participants got back from their walk, they collaborated on a story-telling task adapted from past research [3], which was our primary measure of creativity in Study 1. Researchers gave each group a sheet of paper that had a one-sentence prompt on it, which read “While hiking in the woods, a young couple was startled when they heard something rustling in the bushes behind them.” Researchers instructed participants to take turns continuing to write the story by passing the sheet of paper from group member to group member around the table and each writing one sentence at a time. Together, these individual sentences would form a complete story. This process repeated for ten minutes. At the end of the ten minutes, the researcher collected the story from the participants.

Two coders independently rated each story on creativity and complexity on a scale from 1 (Not at all original/complex) to 5 (Extremely original/complex). Coders were blind to the condition (synchrony vs. control) that each story came from. Stories were simply identified by group numbers during coding so that researcher bias could not affect coding decisions. When rating creativity, coders were told to consider the presence of atypical characters and storylines that demonstrated innovation on common literary tropes (Krippendorff’s alpha = .76). When rating complexity, coders were told to consider qualities such as the presence/absence of dialogue, the number of settings in which the story took place, and the linearity of the story’s plot (Krippendorff’s alpha = .58). Coders’ scores were averaged together to create a composite score for creativity and for complexity for each story. We also used Linguistic Inquiry and Word Count [4] to calculate the length (word count) of each story. Excerpts from four stories rated highest and lowest on creativity are included in Table S1.

**Table S1. Representative Story Excerpts**

| **Creativity Ranking** | **Excerpt** |
| --- | --- |
| 5.0 out of 5.0 | It was Darth Vader. They were filming a new movie. He was looking for his long lost lover. |
| 5.0 out of 5.0 | Approaching to investigate, they discovered a dolphin flopping on the dirt and leaves, gasping in pain, a samurai sword stuck in its flank. The couple looked around to see if anyone was around. They looked all over the place and noticed a leprechaun sitting on a tiny bench. |
| 1.5 out of 5.0 | They turn around, and to their surprise, they find a huge black bear behind them. Startled by the bear, the couple immediately ran away, but the bear followed in pursuit. They ran as fast as they could, passing through curves in the trail and steep hill when Jenny tripped and fell. |
| 1.5 out of 5.0 | They looked behind them, and did not see anything, so they continued with their hike. After several minutes of hiking they heard the noise again. Being an adventurous couple, they decided to follow the noise. |

*Note.* The first two studies are two of the highest rated for creativity, and the last two stories are two of the lowest rated for creativity. Each excerpt contains the first three sentences of the story.

**Study 2**

**Participants & Methods**

Our second study recruited a total of 278 undergraduate participants (71.2% female, *M*_age_ = 20.50, *SD*_age_ = 4.17) who made up 80 groups of either three or four members. Each group was randomly assigned to either a synchrony condition or a control condition. We manipulated synchrony through a chanting task adapted from past research [5] that required the group to either chant the same one-syllable words as each other (synchronous) or different one-syllable words from each other (asynchronous) for six minutes.

Following this manipulation, we measured group dissent using the ACME group decision making task [6], a hidden profile task that requires a group to reach a decision when one person in the group has different, more complete information from the rest of the group members. Crucially, the group is not aware that one of the members has different information, creating the opportunity for dissent within the group. In this task, participants chose where they would like to sit around a table and were told to imagine that they were part of a company that must decide to acquire one of three other companies: Company A, Company B, or Company C. They were given paper packets with information on each company that clearly favored Company B, but one participant in each group (termed the “minority participant”) was given extra information which clearly showed that Company A was the best choice. This role was randomly assigned during each session by always handing the packet with extra information to the person sitting in the front right seat in the room. The group would not be able to reach the correct answer – Company A – without dissenting input from this participant [6].

Trained research assistants sat in on the group negotiation sessions and recorded information on qualities of the discussion relevant to dissenting input. They coded for the presence of “information pooling,” or whether or not the participant with extra information shared this unique information with the rest of the group, with a binary 0/1 response (a value of 1 represents the presence of information pooling). They also coded for whether or not the participant with extra information made an argument in favor of their unique opinion during the discussion using the same binary 0/1 response (a value of 1 indicates the presence of an initial argument). Lastly, research assistants coded the total number of times the participant with extra information repeated their argument, including the first mention.

**References**

1. Boyd R, Silk JB. How humans evolved: Seventh edition. W. W. Norton & Company; 2014.
2. Wiltermuth SS, Heath C. Synchrony and cooperation. Psychol Sci. 2009 Jan 1;20(1):1-5.
3. Amabile TM, Hennessey BA, Grossman BS. Social influences on creativity: The effects of contracted-for reward. J Pers Soc Psychol. 1986;50(1):14–23.
4. Pennebaker JW, Francis ME, Booth RJ. Linguistic inquiry and word count: LIWC 2001. Austin, TX: LIWC.net; 2001.
5. Reddish P, Fischer R, Bulbulia J. Let’s dance together: synchrony, shared intentionality and cooperation. PLoS One. 2013 Aug 7;8(8):e71182.
6. McLeod PL, Baron RS, Marti MW, Yoon K. The eyes have it: Minority influence in face-to-face and computer-mediated group discussion. J Appl Psychol. 1997 Oct;82(5):706.
